# Argonaute and small RNAs in the nucleus: Mediators of gene silencing and activation

**DOI:** 10.1016/j.omtn.2026.102938

**Published:** 2026-04-22

**Authors:** Evgeniia Shcherbinina, Michelle Fong, Annabelle Biscans, Aishe A. Sarshad

**Affiliations:** 1 Department of Medical Biochemistry and Cell Biology, Institute of Biomedicine, University of Gothenburg, 40530 Gothenburg, Sweden; 2 Wallenberg Centre for Molecular and Translational Medicine, University of Gothenburg, 40530 Gothenburg, Sweden; 3 Nucleic Acid Therapeutics Department, Discovery Sciences, BioPharmaceuticals R&D, AstraZeneca, 43138 Gothenburg, Sweden; 4 Ribocure Pharmaceuticals AB, 43153 Gothenburg, Sweden

**Keywords:** MT: non-coding RNA, small interfering RNAs, microRNA, small activating RNA, argonaute protein, RNA interference, RNA activation, oligonucleotide therapeutics, RNA therapeutics

## Abstract

RNA molecules are dynamic regulators of gene expression, with duplex RNAs such as siRNAs and saRNAs functioning as versatile effectors that can both silence and activate gene expression through Argonaute (AGO) proteins. While cytoplasmic RNA interference (RNAi) is well established, the mechanisms and outcomes of nuclear RNAi and RNA activation (RNAa) are only emerging. This review integrates recent discoveries on nuclear RNAi and RNAa, outlining how siRNAs can target nuclear long noncoding RNAs, promoter-associated transcripts, and pre-mRNAs to mediate silencing, transcriptional repression, or alternative splicing. Conversely, saRNAs can recruit AGO2 and transcriptional cofactors to activate gene expression through chromatin remodeling and RNA polymerase II engagement. Beyond their mechanistic roles, we highlight the growing therapeutic potential of duplex RNAs, which can be harnessed to selectively silence or activate gene expression, offering new strategies for RNA-based precision medicine.

## Graphical abstract

**Figure:**
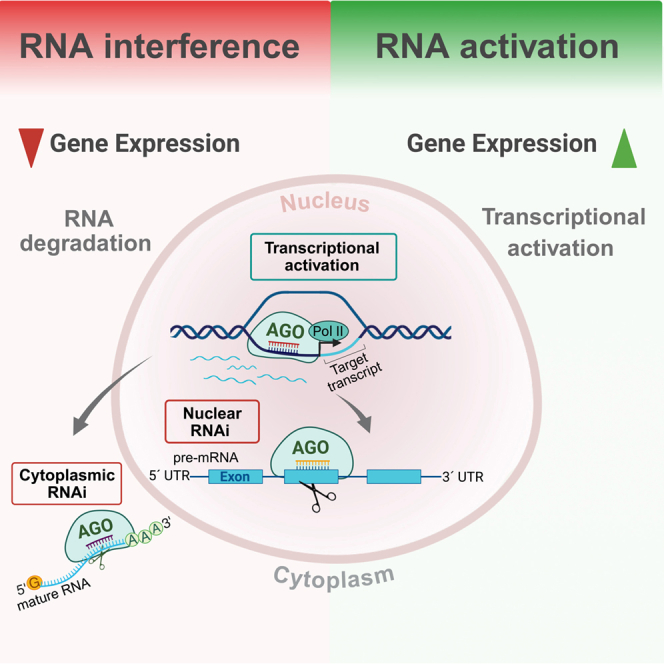

## Teaser

Nuclear Argonaute-small RNA complexes extend RNA regulation beyond the cytoplasm, enabling gene silencing and activation. While mechanistic understanding remains limited, these pathways reveal a versatile layer of gene regulation with significant therapeutic potential for precise modulation of gene expression.

## Introduction

RNA molecules have long been regarded as passive carriers of genetic information, simply bridging DNA and protein in the central dogma. Today, they are recognized as versatile regulators of gene expression, essential structural components of cellular architecture, and catalysts of biochemical reactions, among many other roles.^1^

**Figure 1:**
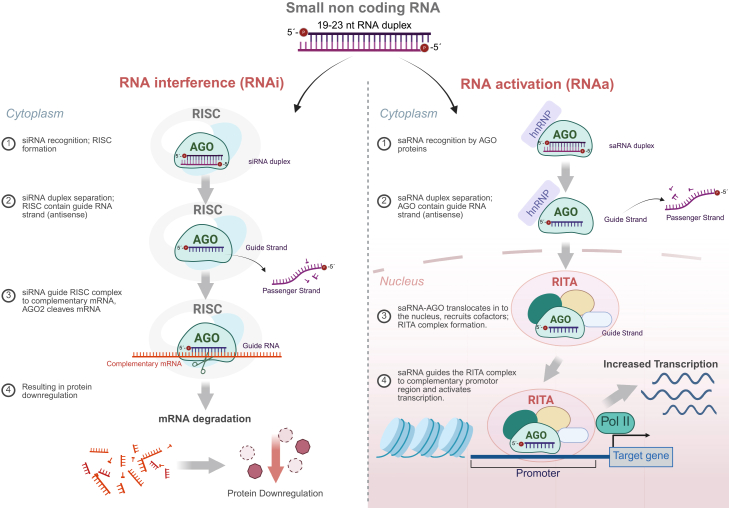
Schematic representation of the general modes of action of RNAi and RNAa RNAi occurs in both the cytoplasm and nucleus: miRNA-mediated RNAi recruits the RISC complex to repress target RNAs, whereas siRNA-mediated RNAi direct AGO2 to cleave complementary targets. RNAa occurs in the nucleus, where duplex RNAs directs AGO2 to bind promoter-associated RNAs to facilitate RITA assembly and transcriptional activation.

RNAs exist in a remarkable variety of shapes, lengths, and structural conformations, ranging from short microRNAs (miRNAs) to long noncoding RNAs (lncRNAs) and highly structured ribosomal RNAs (rRNAs).^1^ This structural and functional diversity enables them to regulate virtually every aspect of gene expression. Within this landscape, duplex RNAs represent a particularly intriguing class. While some duplex RNAs are endogenously generated, others can be synthetically introduced into cells, where they engage in gene regulation.^2^ Certain small duplex RNAs are capable of guiding both gene silencing and gene activation, underscoring their dual regulatory potential and highlighting their relevance to both RNA interference (RNAi) and RNA activation (RNAa) mechanisms^3^ (Figure 1).

The dual regulatory capacity of duplex RNAs converges on a single molecular effector—the Argonaute (AGO) protein, which binds small RNAs and directs them to complementary targets to silence or activate gene expression.^4^ In humans, four AGO paralogs (AGO1-4) bind small RNAs but only AGO2 possesses endonuclease (“slicer”) activity capable of directly cleaving RNA targets.^4^ AGO proteins share a conserved domain architecture: an N-terminal domain involved in duplex unwinding and passenger-strand removal; a PAZ domain that anchors the 3′ end of the guide RNA; a MID domain that binds the 5′ phosphate of the guide RNA; and a PIWI domain, structurally similar to RNase H, which mediates cleavage in catalytically active AGOs.^4^^,^^5^

In the canonical RNAi pathway, AGO-bound small RNAs, typically small interfering RNAs (siRNAs), or miRNAs guide, the complex to mRNA targets, leading to translational repression or mRNA destabilization.^6^^,^^7^ In contrast, RNAa involves small RNAs that target promoter-associated or nascent transcripts to activate gene expression, often through chromatin remodeling or transcriptional initiation.^8^^,^^9^^,^^10^ In this review, we explore how duplex RNAs function within the nucleus of human cells, a relatively uncharted territory for RNAi and the emerging field of RNAa and try to address a central question: how does AGO, using the same conserved domains and guide principles, distinguish between classical RNA-guided silencing and transcriptional activation?

## RNAi

### Endogenous miRNA- and AGO-mediated post-transcriptional gene silencing

The best-characterized example of small RNA-guided gene regulation is the miRNA pathway, which directs AGO-dependent post-transcriptional gene silencing in eukaryotic cells. Because synthetic small RNAs exploit many of the same molecular components, it is important to first outline the canonical biogenesis and function of miRNAs. miRNAs are transcribed by RNA polymerase II as long primary transcripts (pri-miRNAs), which are capped and polyadenylated like mRNAs.^11^ In the nucleus, the microprocessor complex composed of the RNase III enzyme Drosha and its cofactor DGCR8 cleaves pri-miRNAs to release ∼70 nucleotide precursor miRNAs (pre-miRNAs).^11^ These hairpin precursors are exported to the cytoplasm by Exportin 5, where they are further processed by another RNase III enzyme, Dicer, into ∼22 nucleotide duplexes. One strand of the duplex, the guide strand, is then incorporated into an AGO protein to form the miRNA-induced silencing complex (miRISC).^11^

Once loaded, miRNAs direct miRISC to target mRNAs, primarily through imperfect base pairing, particularly involving the seed sequence (nucleotides 2–8 of the miRNA). Unlike siRNAs, which usually induce AGO2-mediated cleavage due to near-perfect complementarity, miRNAs typically repress gene expression by recruiting effector proteins such as TNRC6 and the CCR4-NOT deadenylase complex.^12^ This results in mRNA deadenylation, decapping, and degradation, or in translational repression when transcripts remain intact.^12^ Through this combination of biogenesis and action, miRNAs function as versatile regulators that fine-tune gene expression and shape complex regulatory networks, often simultaneously modulating dozens to hundreds of targets within the cell.

### Non-canonical miRNA- and AGO-mediated post-transcriptional gene silencing

In eukaryotes, nuclear RNAi was first described in plants, where small RNA pathways repress target genes at the transcriptional level by directing epigenetic chromatin modifications, such as through DNA methyltransferases or histone.^13^ Later, in the early 2000s, nuclear RNAi pathways were identified in *C. elegans*, showing co-transcriptional gene silencing by inhibiting RNA polymerase II elongation or guiding epigenetic modifications.^13^ For many years in humans, RNAi was thought to occur exclusively in the cytoplasm, consistent with the enrichment of AGO proteins, TNRC6, and other cofactors in cytoplasmic foci such as P-bodies.^14^ Moreover, in 2006, Ohrt et al. demonstrated active exclusion of siRNA from the nucleus by Exportin 5,^15^ and in 2002, Zeng and Cullen showed that RNAi could cause target mRNA degradation during nuclear mRNA export but not when the mRNA was restricted to the nucleus.^16^ However, emerging data suggests that the core components of RNAi are not only restrictive to the cytoplasm but also expands into mammalian cell nuclei. AGO proteins and cofactors such as TNRC6A/B have been detected within the nuclear compartment,^17^^,^^18^^,^^19^^,^^20^ with Importin 8 (IPO8) implicated in mediating the import of AGO-containing complexes.^21^^,^^22^ Nishi et al. further demonstrated that TNRC6A possesses intrinsic nuclear localization and export signals, establishing it as a nuclear-cytoplasmic shuttle that facilitates AGO2 translocation.^23^

Although the precise pathways by which duplex RNAs enter the nucleus remain unclear, recent work clarifies a stress-responsive mechanism: under cellular stress, miRNAs (as well as siRNAs, discussed in the following sections, and oligonucleotide therapeutics) form a stress-induced response complex (SIRC)—which includes AGO proteins (AGO1/AGO2), YBX1, CTCF, FUS, SMAD proteins, and others—that actively shuttles them into the nucleus. This shuttling is enhanced during stress conditions such as arsenite exposure, resulting in increased nuclear localization and enhanced siRNA- or oligonucleotides-directed modulation of nuclear RNA processes (e.g., splicing).^24^

Additional evidence suggests that specific sequence motifs may contribute to nuclear localization of miRNAs. Hwang et al. demonstrated that a 3′-terminal AGUGUU hexanucleotide motif can function as a transferable nuclear localization signal, promoting nuclear accumulation of miRNAs that contain it, whereas its deletion abolishes this activity.^25^ Such motifs may represent a general mechanism for selective miRNA nuclear import, potentially acting in concert with importin-dependent transport pathways.^26^

In the nucleus AGO—miRNA complexes bind target transcripts according to the same seed—complementarity rules used in the cytoplasm but the available target space is expanded to include introns, coding sequences (CDSs) and nascent pre-mRNAs in addition to 3′ UTRs nucleus.^27^^,^^28^ PAR-CLIP and related crosslinking and immunoprecipitation approaches show that a large fraction of nuclear AGO binding maps to intronic regions and CDS, consistent with co-transcriptional engagement of pre-mRNAs and broader target repertoires in the nucleus.^27^ Functionally, nuclear AGO-miRNA complexes can decrease target RNA abundance by recruiting the CCR4-NOT deadenylation/degradation machinery and thereby mediate post-transcriptional silencing within the nuclear compartment.^27^^,^^29^ Beyond post-transcriptional decay, nuclear miRNAs and associated proteins have been implicated in transcriptional regulation—both silencing and activation at promoters—and in the modulation of alternative splicing, suggesting mechanistic coupling between miRNA activity and chromatin/transcriptional states.^29^^,^^30^ Subnuclear localization patterns (for example, nucleolar enrichment of some miRNAs) and dynamic relocalization upon stress indicate regulated storage or specialized nuclear functions for subsets of miRNAs.^29^^,^^31^ Finally, the composition and apparent size of nuclear RNA inducing silencing complex (RISC) can differ from cytoplasmic RISC (nuclear RISC is often biochemically distinct and, in some assays, smaller), implying that nuclear silencing may employ a tailored protein complement and distinct biochemical properties while still relying on canonical miRNA-seed recognition and effector modules.^29^^,^^32^

The detailed study of the endogenous miRNA pathway has established the fundamental principles of AGO-guided gene regulation. These principles have been successfully utilized through synthetic siRNAs to achieve potent gene silencing in both biomedical research and therapeutic applications. More recent discoveries of nuclear miRNA-mediated gene regulation have also sparked interest in synthetic RNAs targeted to the nucleus. In the following sections, we present the growing evidence for nuclear RNAi and RNAa mediated by siRNAs and saRNAs, respectively, and highlight their therapeutic potential. Finally, we briefly discuss emerging data on miRNA-mediated transcriptional activation in transcriptional activation through miRNAs section.

### siRNA- and AGO-mediated post-transcriptional gene silencing

siRNAs were first demonstrated to work in mammalian cells in 2001 by Elbashir et al.^33^ Before this, RNAi had been well established in plants and invertebrates but attempts in mammalian cell culture with long double-stranded (ds) RNAs had failed because they triggered a strong, non-specific antiviral interferon response. The breakthrough came when Elbashir et al. showed that short 21–22 nucleotide duplex RNAs could bypass the interferon system while still mediating potent, sequence-specific gene silencing.^33^

siRNAs are short non-coding dsRNA molecules, about 21 nucleotides long, with two-nucleotide 3′ overhangs on each strand. The duplex consists of a sense strand (matching the target mRNA) and an antisense strand, which guides the silencing process.^4^^,^^34^ Although siRNAs can be found endogenously in certain mammalian cell types, particularly in oocytes, spermatogenetic cells, and embryonic stem cells,^35^ they are most often chemically synthesized and delivered experimentally to achieve targeted gene silencing.^33^ Once inside the cell, the double strands of the siRNA duplex are separated, the sense strand is degraded, and the antisense strand is incorporated into the RISC. RISC complexes use the antisense strand as a guide to recognize perfect or near-perfect complementary sequences in target mRNAs.^4^ Upon target recognition, AGO2 proteins, the catalytic component of RISC, cleaves the mRNA between the 10th and 11th nucleotide from the 5′ terminus of the antisense strand.^36^ This cleavage event marks the mRNA transcript for degradation, effectively preventing translation, and thereby reducing expression of the encoded protein. Through this mechanism, siRNAs provide a highly potent and specific, post-transcriptional means of regulating gene expression in mammalian cells. Because of the efficiency of siRNAs, researchers use these small RNAs both as a powerful tool to study gene function, by selectively “knocking down” genes of interest and as a therapeutic strategy to silence disease-causing genes.^37^

### Mechanisms of action of nuclear-targeting siRNAs

#### siRNA-mediated silencing of nuclear lncRNAs through the canonical RNAi pathway

**Figure 2:**
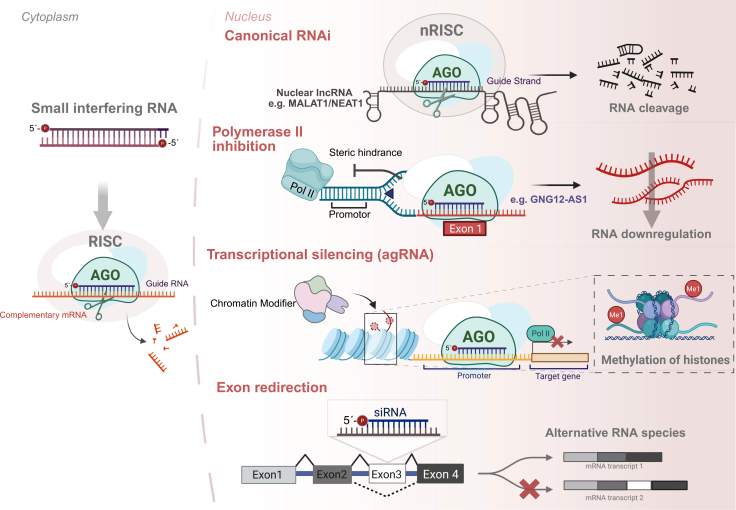
Schematic representation of known siRNA mechanisms in nucleus of mammalian cells siRNA in the nucleus can cause cleavage of nuclear lncRNAs by AGO2 protein via canonical RNAi pathway. Binding of an siRNA to the transcription start site in exon 1 interferes with polymerase II binding, preventing the synthesis of GNG12-AS1 transcript. Promoter targeting siRNAs (agRNAs) direct RNAi machinery to DNA regulatory regions, which cause gene transcription silencing through interaction with chromatin-modifying enzymes. Finally, siRNAs can modulate alternative splicing in the nucleus.

After showing the presence of key RNAi factors in cell nuclei, Gagnon et al. investigated the efficacy of siRNA against nuclear targets.^32^ In HeLa cells treated with siRNAs targeting lncRNAs MALAT1 (metastasis-associated lung adenocarcinoma transcript 1) or NEAT1 (nuclear-enriched abundant transcript 1), their expression levels were measured in different subcellular compartments 72 h after transfection. They observed significant reduction in MALAT1 and NEAT1 transcript levels in all studied compartments: cytoplasm, nucleoplasm, and chromatin. Using 5′ RACE assay, they showed that MALAT1 is cleaved at the predicted AGO2 cleavage site in the cytoplasm, nucleoplasm, and chromatin, and an *in vitro* cleavage assay confirmed that AGO2 can cleave target transcript in both cytoplasmic and nuclear fractions.^38^ Taken together, Gagnon et al. showed that siRNAs can target nuclear transcripts in mammalian cells via AGO2-mediated, site-specific cleavage, resulting in nuclear RNA reduction comparable to the canonical cytoplasmic RNAi mechanism Figure 2. Several subsequent studies further confirmed the ability of siRNAs to target MALAT1, demonstrating that siRNA-mediated knockdown has been successfully applied to investigate its role in various diseases, including cancer, consistently yielding significant reductions in MALAT1 levels.^39^^,^^40^^,^^41^ In addition, effective siRNA-mediated silencing has been reported for other predominantly expressed nuclear lncRNAs, such as NEAT1, GAS5 (growth arrest-specific 5), MEG3 (maternally expressed gene 3), TUG1 (taurine-upregulated gene 1), and PANDAR (promoter of CDKN1A antisense DNA damage activated RNA), across different cancer types.^42^ In the cited studies, the underlying silencing mechanism was not characterized; instead, siRNAs were utilized as effective experimental tools that reproducibly downregulated nuclear lncRNA targets.

Lenox et al. investigated how different chemical modifications influence siRNA and antisense oligonucleotides (ASOs) efficacy against MALAT1 and NEAT1 lncRNA with different subcellular localization.^43^ ASOs function mainly through two mechanisms: recruiting RNase H1 to degrade the RNA strand of an RNA-DNA duplex, or acting via steric blockade to interfere with processes such as splicing, translation, or RNA maturation without RNA degradation.^44^ Even though they observed that nuclear lncRNAs were downregulated by ASOs with higher efficacy than by siRNAs, they achieved significant downregulation of both MALAT1 and NEAT1 by several siRNAs with different chemistry.^43^

#### siRNAs targeting the beginning of the first exon inhibit RNA polymerase II

siRNAs are more widely being used to knockdown nuclear-specific targets; however, relatively few studies have examined the underlying mechanisms of how silencing occurs in the nuclear context, making it unclear whether the effects arise from canonical AGO2-dependent RNAi or from alternative nuclear pathways. Stojic et al.^45^ described an interesting mechanism of nuclear silencing by siRNAs targeting the lncRNA GNG12-AS1 (gamma subunit of G protein 12-antisense RNA 1). GNG12-AS1 is a stable, chromatin-associated transcript, often accumulating near its site of transcription in distinct nuclear foci. The researchers employed multiple siRNAs targeting different exons of GNG12-AS1 and discovered that while most siRNAs achieve post-transcriptional silencing, a specific siRNA-targeting exon 1 in proximity to the transcription start site (TSS) exerted a transcriptional silencing effect.^45^ This siRNA recruited AGO2 to the lncRNA’s promoter region, inhibited RNA polymerase II binding, and suppressed nascent GNG12-AS1 transcription without altering repressive chromatin marks (Figure 2). This approach demonstrated that carefully designed siRNAs can differentially modulate nuclear lncRNAs, enabling researchers to distinguish effects on transcription from those of the mature RNA molecule.^45^

#### Promoter-targeting siRNAs, agRNAs

Several studies presented evidence of siRNA ability to play a role in transcriptional regulation.^38^^,^^46^^,^^47^^,^^48^ They showed that promoter-targeted siRNAs (also called antigene RNAs [agRNAs]) can silence gene transcription by directing the RNAi machinery to regulatory regions of DNA. siRNAs were designed to be complementary to promoter regions of target genes, and upon binding, the guide strand of the siRNA recruited AGO2 to these nascent promoter transcripts. This recruitment initiated transcriptional gene silencing through interactions with chromatin-modifying enzymes or other mechanisms.^38^^,^^46^^,^^47^^,^^48^

Cho et al. demonstrated that agRNAs targeting the androgen receptor (AR) promoter recruits AGO2 together with the histone methyltransferase SETDB1 (SET domain bifurcated histone lysine methyltransferase 1), leading to the repressive histone mark H3K9me3 at the AR promoter. Additionally, PRC2-EZH2 (polycomb repressive complex 2 with its catalytic subunit EZH2) joins AGO2-SETDB1 RNA-induced transcriptional silencing complex and adds H3K27me3 to the AR promoter. These changes in histone modifications remove RNA polymerase II from the promoter, preventing AR transcription.^41^

Hawkins et al. showed that dsRNAs specific to ubiquitin C (*UbC*) gene promoter region resulted in the silencing of *UbC* transcription. Silencing initiation was achieved via site-specific histone methylation, which required the presence of an *UbC* promoter-associated RNA (pRNA), HDAC-1, DNMT3a, and AGO1. Meanwhile, to maintain *UbC* silencing, DNMT1 and possibly DNMT3a proteins were required.^47^ Similarly, Roberts et al. demonstrated that siRNAs complementary to myostatin promoter caused silencing of myostatin gene expression dependent via histone deacetylation as well as they detected pRNAs at myostatin locus.^47^

Alternatively, Napoli et al. showed that siRNA targeting c-Myc promoter causes c-Myc transcriptional silencing via distinct mechanism.^47^ They did not observe any changes in epigenetic marks upon siRNA binding; instead they showed that c-Myc promoter-targeting siRNA disrupted transcription initiation via promoter-specific RNA-directed transcriptional interference (RdTI). RdTI is AGO2-dependent and occurs when siRNA binds to pRNAs as it is transcribed upstream of the c-Myc TSS, thereby preventing the assembly of the transcription pre-initiation complex.^47^

Collectively, these findings show that siRNAs targeting promoter regions can regulate gene expression at the transcriptional level, distinct from their cytoplasmic mRNA-cleaving role (Figure 2). In the nucleus, such siRNAs act through a pathway in which sequence-specific recognition of pRNAs directs AGO2 and chromatin-modifying factors to establish an epigenetically remodified state.

#### siRNAs regulate alternative splicing

Several studies from the Corey lab explored the nuclear potency of siRNAs to redirect alternative splicing of target genes.^49^ They demonstrated that siRNAs could redirect alternative splicing in three distinct systems by designing duplex RNAs complementary to sequences near aberrant splice sites. First, in an engineered HeLa cell line, which expresses luciferase gene containing a defective splice site (HeLa pLuc/705), siRNA duplex restored correct exon inclusion leading to increased luciferase activity. Second, in the SMN2 gene—relevant to spinal muscular atrophy—siRNAs promoted inclusion of exon 7, shifting splicing toward the full-length, functional SMN protein isoform. Third, in the dystrophin gene, implicated in Duchenne muscular dystrophy, siRNAs targeting sequences in a mutated exon induced exon skipping, allowing production of a partially functional dystrophin transcript. In all cases, AGO2 was shown to be essential for splicing correction, and the siRNAs were modified to disrupt AGO2’s cleavage activity, thereby shifting their role from degrading mature transcripts in the cytoplasm to modulating splicing events within the nucleus.^49^

Although RNAi has long been associated exclusively with gene silencing, accumulating evidence has revealed a more complex and versatile role for small duplex RNAs in gene regulation. Remarkably, the same class of molecules capable of repressing transcription can, under certain conditions, activate it.^8^^,^^17^ This phenomenon, termed RNAa, reflects a paradigm shift in our understanding of small RNA biology, demonstrating that duplex RNAs are not limited to post-transcriptional repression but can also promote transcriptional gene activation within the nucleus.

## RNAa

### saRNAs are an emerging class of small RNAs

saRNAs represent a class of duplex RNAs that positively regulate gene expression through RNAa. Similar to siRNA, saRNAs are typically 19–21 nucleotides in length with 2-nucleotide 3′ overhangs and assemble with AGO2 to form ribonucleoprotein complexes.^8^ However, they diverge fundamentally in their mechanisms of action once AGO2 is loaded with the guide strand of the small RNA. Instead of targeting mRNAs, they recognize promoter regions through complementarity between the antisense guide strand and sequences within or near the gene promoter, thereby activating transcription.^8^^,^^17^

The phenomenon of RNAa was first reported in 2006 when Li et al. demonstrated that synthetic siRNAs targeting the promoter regions of E-cadherin, p21WAF1/CIP1 (p21), and VEGF in human prostate cancer cell lines (PC-3 and DU145) induced upregulation of the corresponding mRNAs above basal levels.^22^ At first, the phenomenon raised debate over whether it represented a new pathway, or simply an extension of RNAi but RNAa was soon independently confirmed by Janowsky et al. in 2007, who showed RNAa-mediated upregulation of the progesterone receptor (PR) gene.^50^ Subsequent work expanded RNAa biology across systems and species. In 2010, Huang et al. showed that RNAa is a conserved mechanism across mammalian cells, with saRNAs activating transcription in mouse, rat, and primates.^51^ Additional studies have since demonstrated that RNAa activity is an endogenous regulatory mechanism conserved from *C. elegans* to humans.^51^^,^^52^ RNA-activated transcriptions has been implicated in diverse cellular processes, including cell differentiation,^18^^,^^53^ stress responses,^54^ and regulation of developmental genes.^55^^,^^56^ Its capacity to selectively upregulate specific genes has generated substantial interest in therapeutic applications, including the targeted activation of tumor suppressors in cancer and the modulation of gene expression in regenerative medicine. Notably, the first saRNA-based therapeutics has now advanced into clinical trials,^57^ demonstrating the translational potential of RNAa for treating human disease. Despite these advances, key mechanistic questions remain unresolved, including the precise nature of saRNA-promoter interactions, the full complement of cofactors involved, and the molecular rules that determine target specificity.

### Mechanisms of action of nuclear-targeting saRNAs

The precise mechanism of saRNA activation remains unclear, despite the fact that promoter-targeting saRNAs were identified already in 2006.^22^ Current models suggest several potential routes as follows: (1) direct binding to promoter DNA,^58^^,^^59^ (2) interaction with promoter-derived noncoding transcripts,^60^ or (3) cleavage of antisense transcripts complementary to target mRNAs or upstream genes.^61^

The efficacy of saRNA-mediated gene activation appears to be strongly influenced by promoter architecture. In 2016, Wang et al. demonstrated that type 1 promoters, which contain a TATA box usually located about 25–30 bp upstream of the TSS, are particularly responsive to saRNA targeting. Sequences near the TSS, or within the TATA-box centered region, typically spanning 200–1,200 bp upstream, tend to yield higher activation efficiency compared to type 2 promotors.^62^ Consistent with this, most experimentally validated saRNA target sites are located within 1 kb upstream of the TSS and typically exhibits a GC content of 40%–60%.^10^

In 2016, Portnoy et al. provided key insights into the mode of action of saRNAs, revealing that RNAa is carried out by the RNA-induced transcriptional activation (RITA) complex, which includes AGO2, heterogeneous nuclear ribonucleoproteins hnRNPA2/B1, RNA helicase A (RHA), the RNA polymerase-associated protein CTR9, and DEAD-box helicase 5 (DDX5).^8^^,^^63^ The essential role of AGO2 in the initial stages—binding the saRNA guide strand in the cytoplasm and facilitating its nuclear import—has been validated in multiple knockdown experiments.^8^^,^^50^^,^^63^ Several studies using ChIP analysis or siRNA-mediated knockdown have also implicated AGO1 in saRNA function, suggesting that some saRNAs may require other AGO family members for their activity.^17^^,^^60^^,^^63^

Upon cellular delivery, saRNAs are loaded onto AGO2 in the cytoplasm, where the passenger strand is discarded and the guide strand directs the complex to target promoters.^8^^,^^64^ Unlike canonical RNAi, AGO2’s slicer activity is not engaged, allowing transcriptional activation rather than RNA cleavage.^60^^,^^65^ The molecular basis of AGO2’s switch from silencer to activator, however, remains unclear.

**Figure 3:**
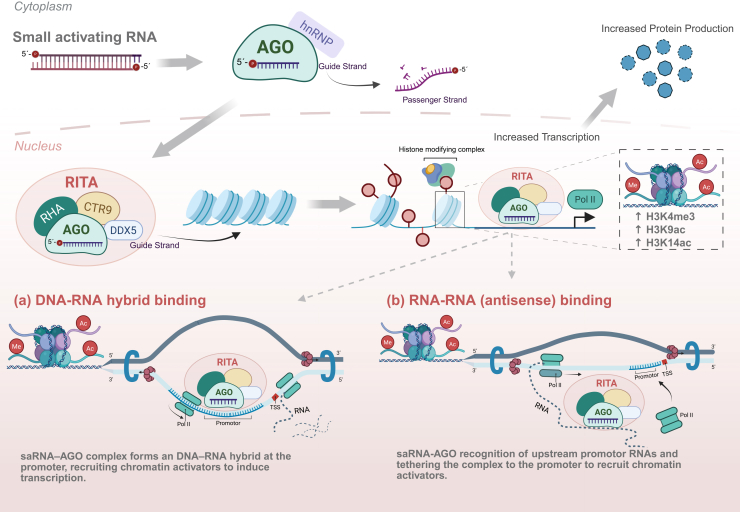
Schematic representation of saRNA-mediated transcriptional activation mechanisms in the nucleus of mammalian cells In the cytoplasm, saRNAs are incorporated into AGO2 proteins, the passenger strand is removed, allowing for an active AGO-saRNA complex formation. After entering the nucleus, the RITA complex is assembled and is proposed to engage regulatory regions through distinct pathways. Binding of saRNAs to complementary nascent promoter-associated transcripts near the transcription start site facilitates recruitment of chromatin-modifying enzymes, promoting an open chromatin configuration and enhancing RNA polymerase II (Pol II) loading. In parallel, promoter-targeting saRNAs (agRNAs) can interact with complementary sequences within regulatory DNA regions, guiding AGO-containing complexes to sites that stimulate transcription by inducing activating histone modifications. Through these mechanisms, saRNAs act as positive regulators of gene expression, driving transcriptional activation rather than repression.

Once at the promoter, AGO2 recruits transcriptional cofactors, including RHA, which is thought to unwind the DNA helix to facilitate saRNA access to its target sites.^8^ RHA is also known to promote transcriptional activation by recruiting basal transcription machinery and altering chromatin structure.^66^ These observations suggest that saRNA targeting may involve recognition of either promoter DNA or nascent transcripts, although this distinction remains unresolved. Portnoy et al. further demonstrates that AGO2 recruits CTR9, a core component of the PAF1 complex (PAF1C), which links transcriptional regulation to chromatin remodeling by modulating RNA polymerse II activity.^8^ CTR9 further facilitates the recruitment of histone-modifying enzymes, including E2/E3 ubiquitin ligases, which drive H2B ubiquitination followed by H3K4 and H3K79 methylation, establishing a chromatin environment that is permissive to transcriptional activation^8^^,^^9^ (Figure 3).

The assembly of these factors at the promoter defines the RITA complex, which directly engages RNA polymerse II and promotes its transition from a paused to an elongating state.^67^ This transition is marked by a decrease in Ser5-phosphorylated RNA polymerse II, characteristic of promoter-proximal pausing, and a concurrent increase in Ser2-phosphorylated RNA polymerse II, indicative of active transcription elongation.^68^ Importantly, the RITA complex lacks several hallmark constituents of cytoplasmic RNAi machinery, including TNRC6, Dicer, and TRBP,^8^ underscoring that the pool of AGO2 involved in RNAa engages a distinct set of nuclear cofactors.

### Transcriptional activation through miRNAs

Recent studies have shown that certain miRNAs can also function as transcriptional activators in the nucleus, a phenomenon termed miRNA-mediated RNA activation (miRNAa). These nuclear miRNAs, often referred to as NamiRNAs, share mechanistic features with saRNAs, including seed complementary and tolerance for mismatches.^69^ Consistent with these similarities, several nuclear-localized miRNAs have been shown to activate gene expression by interacting with promoter DNA or promoter-associated transcripts, thereby functioning in a manner analogous to saRNAs.^69^^,^^70^

The biological implications of miRNAa are significant. In cancer, miRNAa has been shown to modulate key hallmarks of malignancy. For example, miR-21 can interact with the programmed cell death 4 (PDCD4) promoter region in breast cancer cells, decreasing PDCD4 expression and increasing cell proliferation,^71^ while miR-122 has been demonstrated to enhance hepatitis C virus (HCV) replication by binding to the 5′ non-coding region of the viral genome.^72^ Moreover, miR-373 have been reported to activate both E-cadherin, which lead to restoration in epithelial characteristics and potentially reverse epithelial-mesenchymal transition (EMT) in certain cancer cells, and target cold shock domain-containing protein C2 (CSDC2) transcription by targeting their promoters.^73^ Other small RNAs, including miR-744 and miR-1186, have been implicated in upregulation of Ccnb1 expression.^74^

Beyond cancer, miRNAa has also been implicated in developmental pathways and immune regulation, highlighting its broader importance in maintaining cellular homeostasis. For instance, nuclear miRNAs have been shown to influence lineage-specific gene activation during embryonic development and promote transcriptional programs required for stem cell renewal and differentiation.^75^ In the immune system, miRNAa contributes to the regulation of cytokine expression^20^ and T cell activation,^21^ underscoring its role in fine-tuning both adaptive and innate responses. Together, these findings suggest that miRNAa functions as a versatile layer of gene regulation extending well beyond oncogenic contexts.

## Therapeutic potential of nuclear-targeting duplex RNAs

Small duplex RNA-based therapeutics has reshaped the landscape of molecular medicine, offering unprecedented control over gene expression. Within this field, siRNAs and saRNAs represent two complementary approaches—gene silencing and gene activation—that together hold vast therapeutic potential. While siRNA therapy has achieved clinical maturity,^37^ saRNA therapeutics are now emerging as the next frontier in RNA-based regulation.^76^

### siRNA therapeutics

siRNA therapeutics have emerged as a promising class of precision medicines, capable of treating diseases at the genetic level^37^ (Table 1). These drugs utilize RNAi to selectively silence target genes, offering a highly specific approach compared with traditional therapies. Over the past few years, seven siRNA-based drugs have been approved by the FDA, demonstrating the clinical potential of this technology.^37^^,^^77^ Patisiran (Onpattro) is among the first approved siRNA therapies, which treats hereditary transthyretin-mediated amyloidosis (hATTR) by reducing the production of the disease-causing transthyretin protein.^37^ Givosiran (Givlaari) followed, targeting acute hepatic porphyria (AHP) to decrease the accumulation of toxic heme precursors. For metabolic disorders, lumasiran (Oxlumo) and nedosiran have been approved for primary hyperoxaluria, addressing the excessive production of oxalate in the liver.^37^ Inclisiran (Leqvio), used for hypercholesterolemia, demonstrates the application of siRNA in cardiovascular diseases by lowering LDL cholesterol through silencing the *PCSK9* gene.^37^ In 2022 vutrisiran (Amvuttra) was approved for adults with hATTR amyloidosis with polyneuropathy, targeting transthyretin (TTR) gene. In 2025, the first siRNA-based drug targeting hemophilia A and B, fitusiran (Qfitlia), was approved by the FDA.

**Table 1:** List of approved siRNA-based drugs and siRNA drug candidates that entered clinical trials as of 2025

Candidate	Target gene	Indication	Stage	Sponsor	Delivery/notes
Patisiran (Onpattro)	TTR	hATTR amyloidosis with polyneuropathy (adults)	approved (FDA 2018; EU 2018)	Alnylam	IV LNP; first approved siRNA; knocks down mutant and wild-type TTR
Givosiran (Givlaari)	ALAS1	acute hepatic porphyria (AHP)	approved (FDA 2019; EU 2020)	Alnylam	SC GalNAc; reduces hepatic ALAS1 to lower ALA/PBG
Lumasiran (Oxlumo)	HAO1	primary hyperoxaluria type 1 (PH1)	approved (EU 2020; FDA 2020)	Alnylam	SC GalNAc; lowers glycolate oxidase to reduce oxalate
Inclisiran (Leqvio)	PCSK9	hypercholesterolemia or mixed dyslipidemia	approved (EU 2020; FDA 2021)	Novartis (originated at Alnylam)	SC GalNAc; twice-yearly dosing; durable LDL-C reduction
Vutrisiran (Amvuttra)	TTR	hATTR amyloidosis with polyneuropathy (adults)	approved (FDA/EU 2022)	Alnylam	SC GalNAc; quarterly dosing; robust serum TTR knockdown
Nedosiran (Rivfloza)	LDHA	primary hyperoxaluria (PH1-PH2 on US label)	approved (FDA 2023)	Lilly (acquired from Dicerna)	SC GalNAc; inhibits hepatic LDHA to reduce oxalate
ALN-KHK	KHK (ketohexokinase)	metabolic dysfunction-associated steatohepatitis (MASH)/NAFLD with fructose pathway involvement	phase 1 (2025 start reported)	Alnylam	SC GalNAc; aims to reduce hepatic fructose metabolism to alleviate steatosis/fibrosis risk
ALN-HTR	HTR (serotonin pathway target; program code placeholder pending disclosure)	pulmonary arterial hypertension (PAH)	phase 1 (2025 FIH)	Alnylam	SC GalNAc; extrahepatic effect via liver-derived mediator; confirm exact gene on disclosure
Zilebesiran combo trials expansion	AGT (angiotensinogen)	hypertension (combination regimens with SGLT2i or ARBs)	new phase 3 combinations (2025)	Alnylam + Regeneron	SC GalNAc; quarterly/biannual dosing; includes cardiovascular outcomes-oriented protocols
SLN124 (randomized expansion)	TMPRSS6 (matriptase-2)	iron-restricted anemias (e.g., thalassemia, polycythemia vera)	new phase 2/3 starts (2025)	Silence Therapeutics	SC GalNAc; increases hepcidin by silencing TMPRSS6; extra trials initiated in 2025
STP707 (systemic oncology)	TGF-β1/COX-2 (dual siRNA)	solid tumors (systemic administration)	phase 1 (2025 systemic FIH)	Sirnaomics	IV LNP/polymeric nanoparticles; extends from local STP705 to systemic dual-target approach
ARO-C3	C3 (complement component 3)	complement-mediated diseases (e.g., IgA nephropathy; geographic atrophy exploratory)	phase 1 (2025)	Arrowhead	SC ligand-targeted siRNA (TRiM platform); hepatic C3 knockdown to modulate complement cascade
ARO-ENaC-2.0	SCNN1A (ENaC alpha)	cystic fibrosis (CF) adjunct or muco-obstructive lung disease	phase 1 (2025 inhaled)	Arrowhead	inhaled formulation targeting airway epithelia; next-gen chemistry addressing prior ENaC program issues
VIR-2218 Next-gen (VIR-2218.2)	HBV transcripts (conserved regions)	chronic hepatitis B (functional cure combinations)	phase 1/2 (2025)	Vir Biotechnology	SC GalNAc; optimized sequence/chemistry; paired with immune modulators in new 2025 trials
JNJ-3989 Expansion cohorts	HBV transcripts	chronic hepatitis B (combination regimens)	new phase 2 starts (2025)	Arrowhead/Janssen	SC TRiM; multi-trigger siRNA; expanded combos with capsid inhibitors/IFN
Zodasiran outcomes trial	ANGPTL3	familial combined hyperlipidemia/ASCVD risk reduction	phase 2/3 (2025 outcomes-oriented)	Arrowhead	SC GalNAc; deep TG and LDL reductions; moving to broader CV endpoints

SC, subcutaneous; i.v., intravenous; TRiM platform, targeted RNAi molecule platform; FIH, first in human.

The approval of these therapies highlights the advantages of siRNA-based treatments, including high specificity, reduced off-target effects, and potential for long-lasting therapeutic outcomes. However, challenges remain in optimizing delivery systems, improving stability, and minimizing immune responses.^78^ Chemical modifications and delivery platforms including N-acetylgalactosamine (GalNac) conjugation have been instrumental in overcoming these barriers, enabling effective delivery to target tissues.^79^

Importantly, current siRNA therapeutics primarily acts on mature cytoplasmic mRNA, where they guide the RISC to degrade target transcripts. This cytoplasmic focus has proven highly effective for diseases involving well-characterized and accessible mRNA targets. Yet, the nuclear transcriptome, including non-coding RNAs, nascent transcripts, and pRNAs, remains largely untapped. Expanding siRNA applications to the nucleus could unlock new therapeutic opportunities, such as modulation of gene regulatory elements, or correction of nuclear-retained pathogenic transcripts. Future developments in delivery technologies—particularly those enabling nuclear entry—may therefore broaden the therapeutic reach of siRNA beyond its current cytoplasmic limitations.

### saRNA therapeutics

Although RNAa is a relatively recent discovery, growing evidence has highlighted the therapeutic potential of duplex RNA-mediated transcriptional activation^22^^,^^57^^,^^76^ (Table 2). Compared to other gene and protein augmentation strategies, such as plasmid-based gene therapy, CRISPR-mediated genome editing, mRNA therapeutics, or protein replacement,^80^ saRNAs offer distinct advantages. They are small and compact, easier to deliver, cost-efficient to manufacture, and, importantly, do not introduce permanent genomic alterations, thereby reducing the risks associated with DNA-based interventions.^25^^,^^56^ At the same time, saRNAs face challenges distinct from siRNA therapeutics. A central hurdle is the precise design of saRNA duplexes, such that their sequence and chemical architecture favor nuclear promoter targeting and transcriptional activation while minimizing engagement with canonical cytoplasmic RNAi pathways that could otherwise lead to off-target transcript silencing or mRNA degradation.^81^

**Table 2:** List of saRNA drug candidates

Candidate	Target gene	Indication	Stage	Sponsor	Delivery/notes
MTL-CEBPA	CEBPA	advanced hepatocellular carcinoma (HCC); solid tumors	phase 1/2 (clinical)	MiNA Therapeutics	liposomal saRNA; first in human saRNA; phase 2 in HCC; combo with pembrolizumab
RAG-01	CDKN1A (p21)	non-muscle-invasive bladder cancer (NMIBC)	phase 1 (clinical)	Ractigen Therapeutics	intravesical LiCO delivery; FDA fast rrack; early phase 1 responses
RAG-18	UTRN (utrophin)	Duchenne/Becker muscular dystrophy	preclinical	Ractigen Therapeutics	systemic LiCO™ lipid-conjugated delivery; FDA rare pediatric disease designation
HNF4A saRNA	HNF4A	NAFLD/NASH, metabolic disease	preclinical	MiNA Therapeutics/academic collab	dendrimer and GalNAc delivery; improves metabolic profile in rodent models
MTL-STING	TMEM173 (STING)	oncology/immuno-oncology	preclinical	MiNA Therapeutics	saRNA upregulates STING pathway; oncology immunotherapy approach
SIRT1 saRNA	SIRT1	metabolic syndrome/inflammation	preclinical	MiNA Therapeutics	*in vitro*/*in vivo* activation reduces inflammatory responses; patents filed
HbF/HBG saRNA	HBG (fetal hemoglobin)	sickle cell disease/β-hemoglobinopathies	preclinical	MiNA Therapeutics	liposomal delivery; *in vivo* induction of fetal hemoglobin; preclinical posters

Several RNAa therapeutic candidates have already been developed, with early efforts concentrated on cancer and more recent studies expanding to other disease areas through both preclinical and clinical investigations^56^^,^^57^^,^^63^^,^^78^ In 2014, MiNA Therapeutics introduced the first in class saRNA therapeutic, MTL-CCAAT/enhancer-binding protein alpha (CEBPA), formulated in lipid nanoparticles to upregulate CEBPA,^82^ a transcription factor essential for hepatic and myeloid functions and implicated in tumor suppression.^83^ MTL-CEBPA entered its first human clinical trial in 2016 for advanced hepatocellular carcinoma (phase 1, NCT02716012), where 38 patients received i.v. MTL-CEBPA (28–160 mg/m^2^) and showed an acceptable safety profile with no dose-limiting toxicity or maximum tolerated dose. The preclinical studies also demonstrated synergistic tumor suppression when combined with tyrosine kinase inhibitors (TKIs).^81^ These findings led to ongoing combination trials (phase 1a/1b; NCT04105335) with pembrolizumab, an immune checkpoint inhibitor in patients with advanced solid cancer tumors.

More recently, Ractigen Therapeutics developed RAG-01,^84^ a nanoparticle-formulated saRNA targeting p21 (CDKN1A), a cyclin-dependent kinase inhibitor and key regulator of cell-cycle arrest.^84^ RAG-01 entered 2023 phase 1 testing in Australia, for BCG-unresponsive non-muscle-invasive bladder cancer (NCT06351904) and has been granted FDA fast track designation.^84^ Beyond these, p21-saRNAs have shown strong antitumor activity in xenografted prostate and orthotopic bladder cancer models and in human trials, to date, six patients have received treatment, and preliminary findings indicate good tolerability following the first cycle.^85^

Outside oncology, saRNAs are being investigated as regulators of fundamental biological processes, including apoptosis,^86^ hypoxia, inflammation, and preeclampsia.^78^^,^^87^^,^^88^ Collectively, these advances position saRNAs as a versatile and innovative therapeutic platform with expanding applications, and ongoing research is expected to extend their utility into metabolic, neuromuscular, and other complex diseases.^89^

## Perspective and conclusion

Since the discovery of RNAi in *C. elegans* three decades ago, the progression from a fundamental biological phenomenon to a platform for therapeutic development has been remarkable. However, research exploring RNAi and RNAa remains incomplete, particularly with regard to our understanding of the precise molecular mechanisms by which AGO-loaded small RNAs function—especially within the nucleus. To date, evidence supporting nuclear siRNA activity is derived from a relatively small number of studies, and even fewer reports describe nuclear saRNA-mediated mechanisms. Consequently, many of the proposed models remain provisional, and robust conclusions cannot yet be drawn. Independent validation and further mechanistic studies are therefore essential to fully establish the scope and biological relevance of nuclear small RNA-guided gene regulation. The central question we posed at the start of this review—how AGO, using the same conserved domains and guiding principles, distinguishes between RNA-guided silencing and transcriptional activation—remains unresolved. In the following section, we postulate several modes of action that can guide AGO between silencing, or activation: (1) target-guide paring, (2) subcellular localization of AGO, (3) co-factor selection, or (4) post-translational modifications (PTMs) of AGO.

(1) Target-guide paring: AGO-loaded guides regulate RNAs according to the extent and position of complementarity with their targets.^33^^,^^34^ siRNAs mediate near-perfect pairing across nucleotides ∼2–16, enabling AGO2 slicing activity, in both the cytoplasm and the nucleus.^33^^,^^90^^,^^91^ By contrast, miRNA pairing, across nucleotides 2–8, recruits TNRC6-CCR4-NOT repressive complex (cytoplasm or nucleus) or modulate alternative splicing for repression in the nucleus.^12^ saRNAs are recruiting AGO proteins to promoter-proximal regions influencing chromatin structure and transcriptional machinery.^9^ Therefore, they are designed to most often target DNA sense-strand sequences in the gene promoter region (roughly −1 to −100 bp upstream of the TSS).^9^ Many effective sites lie within −500 to +50 bp relative to the TSS, overlapping transcription factor binding clusters, or CpG-rich segments.^10^ Maintaining a perfect complementarity in the seed region (guide positions g2-g8 relative to 5′ end) and allowing a near-perfect pairing through the central region (g9-g12) are essential to maximize activity.^10^^,^^59^
(2) Subcellular localization of AGO2: where AGO2 meets its guide RNA could be a crucial determinant between activator or silencer functions. AGO2 subcellular localization is dynamic across cancer cell line^92^ and primary cells^27^^,^^93^ and therefore, we hypothesize that the efficacy of a promoter-directed duplex RNA for gene activation, could depend on the presence of nuclear AGO2 protein. We propose that cytoplasmic AGO2 would favor canonical RNAi-mediated gene repression and that enforcing nuclear or cytoplasmic AGO2 localization—including by modulating its nuclear import or export—could determine whether compartmentalization is essential for nuclear gene activation versus cytoplasmic silencing.
(3) Co-factor selection: AGO2-loaded small RNAs intrinsically function through multi-protein assemblies, assembling into either RISC (silencing)^36^ or RITA (activation).^8^ The proteins expressed in each complex (discussed in “endogenous miRNA and AGO-mediated post-transcriptional gene silencing” and “mechanisms of action of nuclear-targeting saRNAs", respectively) largely do not overlap.^7^^,^^8^ Therefore, the availability of co-factors could also determine the outcome. We posit that, for example, depleting TNRC6 should not affect RNAa while preventing miRNA silencing. On the contrary, silencing RITA complex factors should block RNAa but not affect cytoplasmic RNAi. Another interesting consideration still to be evaluated is the minimal RITA complex needed for RNAa.
(4) PTMs of subcellular AGO: numerous reports have shown that PTMs direct AGO function. For example, phosphorylation on serin S824, S828, S831, and S834 inhibit mRNA binding,^94^^,^^95^ while phosphorylation on serin S387 affects localization.^93^^,^^96^ There is no evidence yet that nuclear AGO2 is defined by specific PTMs; however, we hypothesize that PTMs could remodel AGO2’s surface, thereby biasing its interactions, potentially favoring either RISC or RITA. In order to assess this, we first need to systematically evaluate the nuclear PTMome of AGO2 protein and thereafter perform site-directed mutagenesis to evaluate how a specific PTM affects compartments-specific AGO2: small RNA-directed gene regulation.

The choice between target silencing, or activation may depend on one or more of the events discussed previously. Future comparative and mechanistic studies will likely clarify this fundamental aspect of AGO-mediated regulation. Advancing our understanding of the molecular mechanisms and contextual determinants that shape the activity of nuclear-acting duplex RNAs will be key to fully defining their regulatory potential.

## Acknowledgments

This work was funded by the Swedish Research Council (grant no. 2019-01855 to A.A.S.); the 10.13039/501100004063Knut and Alice Wallenberg Foundation (grant no. PAR 2020/228 to A.A.S.), the 10.13039/501100003748Swedish Society for Medical Research (grant no. S19-0019 to A.A.S.), the 10.13039/501100001729Swedish Foundation for Strategic Research (grant no. ID24-0029 to A.A.S. and A.B.), and the 10.13039/501100005760University of Gothenburg. All figures were created with BioRender.com.

## Author contributions

E.S. and M.F., literature review and illustrations; E.S., M.F., and A.A.S., writing – original draft; E.S., M.F., A.B., and A.A.S., writing – review and editing.

## Declaration of interests

A.A.S. is a part-time employee at Ribocure Pharmaceuticals, and A.B. is employed at AstraZeneca.
